# Prediction of recombinant *Mycobacterium tuberculosis* α-crystallin oligomer chaperone activity using polynomial graphs

**DOI:** 10.12688/f1000research.16328.2

**Published:** 2020-07-15

**Authors:** Gautam Krishnan, Utpal Roy

**Affiliations:** 1 Birla Institute of Technology and Science, Pilani, Department of Biological Sciences, Goa Campus, NH17B Bypass, GOA 403726, India

**Keywords:** Alpha-crystallin, Chaperone, Purification, Recombinant, Polynomial

## Abstract

**Background:** Mycobacterial α-crystallin (Acr) is a chaperone that prevents misfolding of proteins when *Mycobacterium tuberculosis* is found in a latent form in the host tissue.

**Methods:** Using insulin as a model substrate and utilizing polynomial graphs, we attempted to predict molecular-level interactions that are a function of the oligomeric state of the recombinant protein. The chaperone activity of the recombinant oligomeric Acr was measured at 60°C with Acr samples obtained before gel filtration chromatography and compared with a gel-filtered sample.

**Results:** The polynomial graphs constructed showed improved molecular coverage of the insulin B chain by the oligomer. The 2 ^nd^ order coefficient is the one that changes with the oligomeric ratio of Acr and improves chaperone activity. Polynomial analysis suggested that it could be a useful parameter to predict chaperone activity for potential *in vitro* batches of *M. tuberculosis* Acr based on the dynamic nature of the association and disassociation of oligomers.

**Conclusions**: The results showed that coverage of insulin B chain improved with higher ratio of 9-mer as compared to lower ratios. This was shown by both simulation plots and actual assay data. The polynomial graphs showed increase in the 2 ^nd^ order coefficient, thus suggesting the important role of oligomerisation in improved molecular coverage of insulin B chain.

## Introduction

Mycobacterial α-crystallin (Acr) is an important protein in latent tuberculosis (TB). When infected by *Mycobacterium tuberculosis*, the host survives the infection, yet in 1 out of 3 cases, the bacteria survive in a hypoxic state ( Sherman *et al.*, 2001). The alternative to *in vivo* studies is to clone and express recombinant Acr in *Escherichia coli* and study its chaperone activity *in vitro* ( Gu *et al.*, 2002; Panda *et al.*, 2017; Yang *et al.*, 1999). Substrates used for past enzymatic studies have included citrate synthase and insulin ( Chang *et al.*, 1996; Gu *et al.*, 2002; Mao *et al.*, 2001; Panda *et al.*, 2017). However, there is no precise molecular means to quantify the biological activity of Acr in terms of molecular interactions.

Acr is active inoligomeric state, and though it is known to act as a trimer of trimers ( Chang *et al.*, 1996) or a dodecamer ( Kennaway *et al.*, 2005; Panda *et al.*, 2017), yet it is difficult to study the mechanism by which it inhibits the thermal aggregation or dithiothreitol (DTT)-induced aggregation of insulin.

In the present study, we analysed recombinant Acr preparations in terms of chaperone activity against the insulin B chain substrate, and explored the possibility of using the polynomial graphs in predicting chaperone activity. A theoretical and mechanistic representation using the real data has been used to render the numerical predictions of activity based on polynomial graphs.

## Methods

### Cloning and expression

The *acr* gene of *M. tuberculosis* H37Rv was directionally cloned using the expression vector pET28a as an N-terminal His tag into pET28a using Nde1 and Xho1 restriction enzymes.(Takara). Expression of the recombinant *acr* was initially done at 50 ml scale, using 1 mM isopropyl β-D-1-thiogalactopyranoside (IPTG) induction (37°C for 3h). The cell pellets obtained were used for the SDS-PAGE (15% acrylamide) to check for the expression of protein. The rest of the sample was sonicated using 10 mM Tris pH 7.0 / 5% glycerol followed by centrifugation at 20,000 g at 4°C for 30 mins. Aliquots of supernatant and pellet obtained were used for the 15% SDS-PAGE gel to observe the localization of protein in supernatant or pellet.

Recombinant Acr was purified using Nickel-NTA agarose. The cell pellet obtained after 0.5–1.0 mM IPTG induction was lysed by sonication. The sonicate was centrifuged at 20,000 *g* for 30 mins at 4°C and the supernatant obtained was allowed to bind to a 3 ml of Nickel-NTA resin. The protein was eluted with a 3-step gradient of 300 mM, 400 mM and 500 mM imidazole in buffer containing 20 mMTris pH 7.0, 300 mM NaCl and 5% glycerol.

 The Nickel-NTA eluted fractions containing the Acr were dialysed against 20mM Tris pH 7.0, 100 mM NaCl and 5% glycerol and subjected to gel filtration chromatography on a Sephacryl-200 Hiprep XK 16/60 Column (Pre-packed 120 ml) using the AKTAPurifier system (GE Healthcare Life Sciences). The column was equilibrated using the same buffer and calibration carried out using BIORAD standards, Molecular weight ranging from 670 kDa to 1.5 kDa. SDS –PAGE analysis was carried out with the first 3 lanes loaded with non-gel-filtered samples and the lanes 5–10 with 2 eluted peaks C1 and C2 of different runs with varying amounts of β-mercaptoethnol (β-ME). A Native-PAGE analysis was carried out for both non-gel-filtered and gel-filtered samples, respectively using 8–16% gradient Tris glycine gel, and bovine serum albumin (Hi-Media; 0.5 mg/ml) as a standard. The oligomer size was estimated using a plot of log molecular weight versus distance migrated and estimated sizes calculated using antilog.

### Chaperone assay using insulin as substrate

Activity of recombinant *M. tuberculosis* Acr was assessed at 60°C using three concentrations (83, 118 and 125 μM) of insulin B chain as a substrate, by adding together Acr along with the insulin B chain. Aggregation of the insulin B chain was initiated by addition of 25 mM DTT and the change in the absorbance was monitored at 360 nm for 20 mins in kinetic mode with 1800 time points of 1 second interval. The assay was carried out at 60°C with His-tag-eluted samples at single point three concentrations of 12, 30 and 37.5 μM labelled as non-gel-filtered samples, and also with a His-tag plus gel-filtered sample at a concentration of 12 μM. The assay volume for 12 μM Acr was 400 microlitre while the other 2 assays were done in assay volume of 250 microlitres. Later assays were done at single-point concentrations of 11 µM and 37.5 µM at 60°C (data not shown).

### Polynomial graph analysis

A set of simulation graphs was plotted in MS Excel 97-2003 assuming different ratios of oligomers and the number of molecules of Acr versus the number of molecules of insulin at 118 μM and the percentage of molecules of insulin B chain covered. The assumption was 4 molar concentrations of 5, 10, 20 and 40 μM and 2 different proportions of 9-mer to 12-mer oligomers; 60% of 9-mer and 10% each of 10- to 12-mer and 20% each of 9-mer to 12-mer. This gave an indication about the trends in percentage insulin B chain covered at two different ratios in scenarios, one with higher amount of nonamer and the second a lower amount of nonamer.

The assay data we obtained was used to calculate the percentage inhibition of insulin aggregation versus percentage of molecules of insulin B chain covered with both monomers and oligomers for non-gel-filtered sample. The assumption made was the molecular size of Acr to be 18 kDa as monomer and further plots were constructed assuming the proportion of 9-mers to 16-mers as obtained from Native-PAGE data. The polynomial plots were used to gain insights into the binding of Acr to the insulin B chain.

## Results

### Expression and activity studies

**Figure 1.**
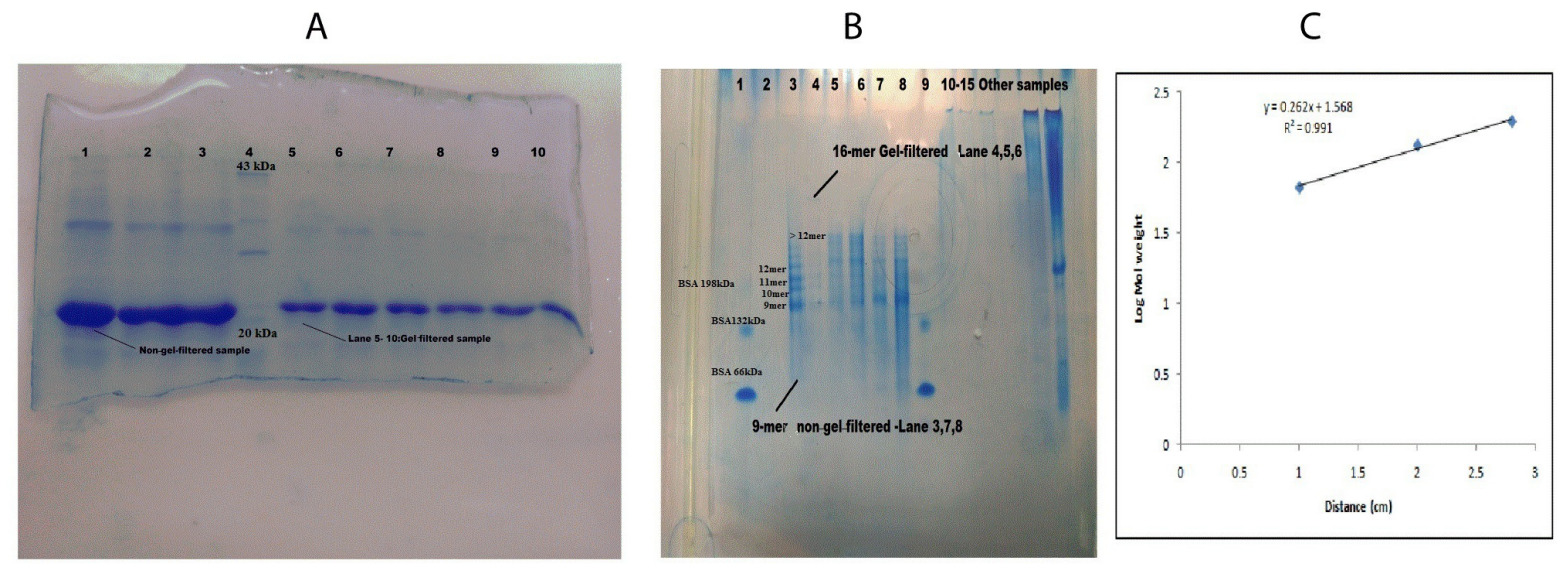
Acr purification and Native-PAGE gel analysis. (Figure 1 changed as recommended by Reviewer 2). ( **a**) SDS-PAGE of gel filtration runs 1 and 2. Lanes 1, 2, 3: Load -1X and 2X β-mercaptoethanol (β-ME); lane 4: marker showing 14, 20, 22, 25, 35, 43, 50, 56, 66 and 80 kDa; lanes 5-7: run 1 C1 1X, 2X and 3X β-ME; lanes 8-10: run 2 C2 1x, 3x and 2x β-ME. ( **b**) Native-PAGE of His-tag-eluted non-gel-filtered samples and gel-filtered elute. Lane 1, 9: BSA control; lane 3: non-gel filtered; lanes 4, 5, 6: gel-filtered; lane 7, 8: non-gel-filtered. ( **c**) Plot of log molecular weight of the different forms of BSA in Native-PAGE. The different sizes of BSA 66, 132 and 198 kDa were plotted against distance migrated and the log molecular weight was plotted in MS excel and the equation displayed and used to calculate oligomer size of non-gel-filtered and gel-filtered samples.

The His-tag purification and gel filtration showed greater than 95% purity as seen in the gel analysis along with upper (higher) molecular weight bands ( Figure 1a). The protein appeared in the void volume suggested a blend of oligomers which was later confirmed by Native-PAGE analysis.

### Native-PAGE analysis

Native-PAGE analysis showed a proportion of 70% of 9-mer and 10% each of 10-mer to 12-mer in the non-gel-filtered (before the gel-filtration chromatography) samples and 40% of 16-mer and 15% each of 9-mer to 12-mer as analysed by ImageJ software ( Figure 1b, c and Table 1).

**Table 1.** Oligomer ratio calculation. The Native-PAGE data was used to calculate the oligomeric sizes and ratios of different bands in non-gel-filtered and gel-filtered samples. Oligomer sizes were calculated using bovine serum albumin (BSA) monomers, dimers and trimers as a reference (66, 132 and 198 kDa) and the log of molecular weights plotted versus the distances travelled in centimetre. From this calculation, molecular weight was extrapolated for the different bands seen in non-gel-filtered samples (band 1, 2, 3, 4) and gel-filtered samples (band 1, 2, 3, 4 and 5).

BSA (Mol. Wt.)	Log Mol wt.	Distance (cm)	Size(kDa)	Monomer(18 kDa)	Fraction of total
66	1.82	1			
132	2.12	2		Monomer	
198	2.29	2.8		18	
				Oligomer size and ratio	Fraction of total
Non-gel-filtered band 1	2.19	2.3	155	9	70
Non-gel-filtered band 2	2.258	2.6	177.13	10	10
Non-gel-filtered band 3	2.297	2.8	198	11	10
Non-gel-filtered band 4	2.332	3	215	12	10
				Ratio of 10-mer to 12-mer	30
				9-mer	70
Gel-filtered band 1		Same as H		9	15
Gel-filtered band 2				10	15
Gel-filtered band 3				11	15
Gel-filtered band 4				12	15
Gel-filtered band 5	2.453	4	283.79	16	40
				Ratio of 10-mer to 16-mer	85
				9-mer	15

### Chaperone activity of Recombinant Acr at higher temperature

**Figure 2.**
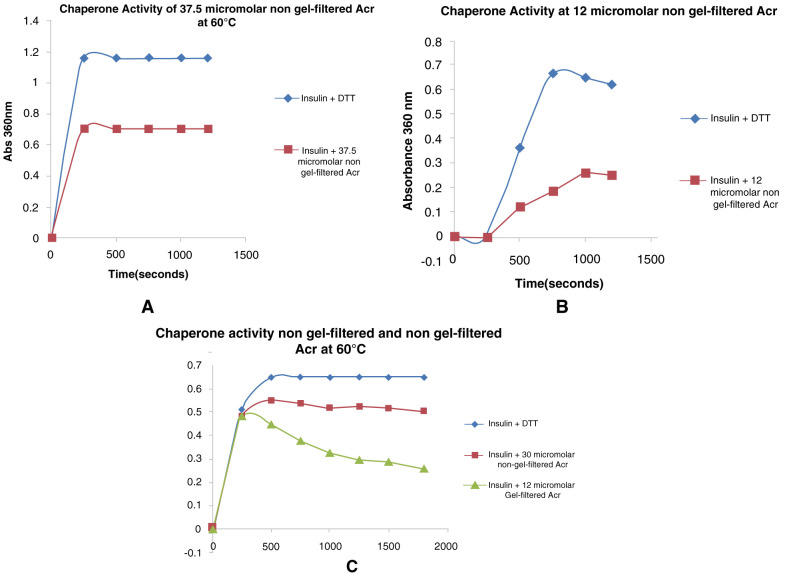
Chaperone Activity of Acr at 60°C. ( **a**) Insulin was assayed at a concentration of 125 µM with α-crystallin (Acr) at a concentration of 37.5 µM in an assay volume of 400 µl by adding Acr to prevent DTT-induced aggregation of B chain. ( **b**) Insulin was assayed at a concentration of 83 µM with Acr added at a concentration of 12 µM in an assay volume of 250 µl. ( **c**) Insulin was assayed at a concentration of 118 µM and using two concentrations of non-gel-filtrered Acr (30 µM) and gel-filtered Acr (12 µM), in an assay volume of 250 µl.

The acr-pET28a construct showed activity against DTT-induced aggregation of insulin. At a concentration of 37 μM of Acr and 125 μM insulin it gave 40% inhibition ( Figure 2a). At a concentration of 12 μMAcr and 83 μM insulin it gave 60% inhibition ( Figure 2b). A separate assay was carried out using both non-gel-filtered and gel-filtered samples at 12 and 30 μM, respectively and insulin at 118 μM concentration ( Figure 2c). There was a difference in activity between both His-tag and gel-filtered samples. The non-gel-filtered samples showed 21.54% inhibition at a concentration of 30 μM, whereas 60% inhibition was achieved by the gel-filtered sample at a concentration of 12 μM Acr.

### Polynomial graph analysis

**Figure 3.**
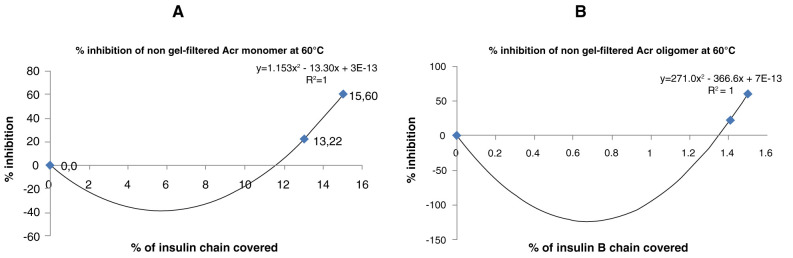
Chaperone activity of Acr non gel filtered versus % of insulin B chain covered. ( **a**) A polynomial plot was used to plot percentage inhibition of non-gel-filtered α-crystallin (Acr) monomers against percentage of of molecules of the insulin B chain covered at 60°C. ( **b**) A polynomial plot was used to plot percentage inhibition of non-gel-filtered Acr oligomer versus the percentage of molecules of insulin B chain covered at 60°C. ( **c**) The percentage inhibition was replotted versus percentage of molecules of insulin B chain at 60°C by calculating the molecules of Acr based on the proportion of oligomers obtained from Native-PAGE analysis at 37°C.

**Figure 4.**
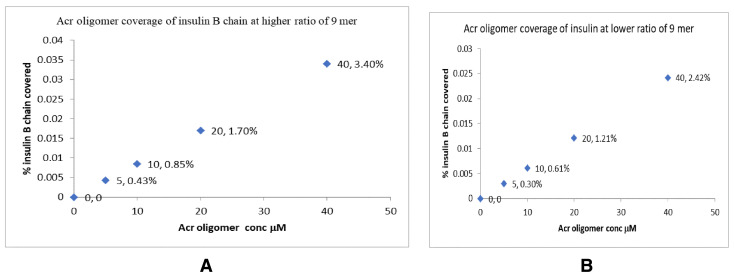
Simulation plots of Acr non gel filtered with different ratios of oligomer. ( **a**) A plot of simulated Acr oligomer assuming 9-mer ratio of 60% and 10% of 10- to 12-mer versus percentage of molecules of insulin B chain covered at 118 µM. ( **b**) A plot of simulated Acr oligomer assuming a 9-mer to 12-mer ratio of 20% versus the percentage of molecules of insulin B chain covered at 118 µM.

Activity of Acr was plotted in terms of percentage inhibition versus percentage of molecules of insulin B chain and polynomial graphs constructed to check for best fit analysis with R ^2 ^greater than 0.95 being the criterion ( Figure 3a, b). The graphs showed 2 ^nd^ order polynomials as the best fit. The coefficient of x ^2^ was higher in the oligomer plot, indicating that oligomerisation decreases the percentage of molecules of the insulin B chain covered. This was performed for the non-gel-filtered samples for both monomer and oligomer, assuming the ratios obtained from Native-PAGE analysis. The simulations showed the proportion of oligomers required to cover a maximum percentage of insulin B chain changed with the proportion of 9-mers with higher proportion of 9-mer covering more of the insulin B chain ( Figure 4a, b).

## Discussion

His-tag purification attempts of the recombinant Acr-producing construct showed the protein obtained was greater than 95% purity in the fractions eluted with 500 mM imidazole. The chaperone activity varied with two different concentrations of Acr, and the plots of monomer versus oligomer obtained showed that oligomerisation helps more molecular coverage the insulin B chain. This was confirmed by the results obtained by the gel-filtered samples that showed higher inhibition than the non-gel-filtered samples. This could be possibly explained by the higher proportion of 10-mer to 12-mer and 40% of 16-mer, which indicates that a higher proportion of oligomers improves chaperone activity.

The generation of polynomial graphs plotted showed an increase in the x ^2^ coefficient of the 2 ^nd^ order graph, which is directly proportional to the amount of oligomers present and can be possibly used to model chaperone activity using insulin as a substrate. These observations suggest that the change in the percentage of molecules of insulin B chain covered is due to the extent of oligomerisation that forms and also varies with the ratios of oligomers directly affecting the activity, suggesting that this is a significant parameter for checking *in vitro* Acr activity.

Polynomial graphs have been used to analyse activity of proteins involving oligomers ( Yeow & Clayton, 2007). Many oligomer proteins function as high-molecular-weight aggregates and polymers, and the mechanism of heat-shock proteins is for that reason, poorly understood ( Mattoo, 2014). This approach of using polynomials can shed insights into the molecular mechanism of oligomers binding to substrates.

## Conclusions

Using polynomial graph analysis, we suggest a predictive tool using percentage inhibition versus insulin B chain covered for *in vitro* Acr preparations that could also be extrapolated for *in vivo* substrates of Acr as a logical outcome for future studies.

## Data availability

**Dataset 1: All raw data for article ‘Prediction of recombinant Mycobacterium tuberculosis α-crystallin oligomer chaperone activity using polynomial graphs’.** Raw, uncropped blots are given in Figure 1a, b. DOI: https://doi.org/10.6084/m9.figshare.12613772 ( Krishnan & Roy, 2020).
