# Supplementary material for: Prediction of recombinant Mycobacterium tuberculosis α-crystallin oligomer chaperone activity using polynomial graphs
Source: F1000Res. 2020 Jul 15;7:1801. Originally published 2018 Nov 15. [Version 2] doi: 10.12688/f1000research.16328.2 (PMC7327723; doi:10.12688/f1000research.16328.2)
Supplement: Peer review history

# Peer review: Reviewer response for version 1

**DOI:** 10.5256/f1000research.17837.r61501

**Published:** 2020-06-30

**Authors:** Nasreen Z. Ehtesham

**Affiliations:** 1 National Institute of Pathology, Safdarjung Hospital, New Delhi, India

The manuscript titled “ **Prediction of recombinant *Mycobacterium tuberculosis *α-crystallin oligomer chaperone activity using polynomial graphs**” by Gautam Krishnan and Utpal Roy demonstrated the relationship between various higher-order oligomers of Acr and its relation to the prevention of aggregation of insulin B chain as a function of its chaperone activity, predicted using a mathematical polynomial graph. Earlier reports showed that Acr exists in multiple higher-order oligomers, which is related to their chaperone functions. Acr is a vital chaperone protein expressed majorly in the stationary phase as well as in hypoxic conditions. It is required for the survival of *M. tb* in macrophages; thus, it is a critical virulence effector. The authors utilize the previously known pieces of information and use of polynomial graphs in the prediction of Acr oligomers and its relation to its chaperone activity. They devised a novel way to predict Acr chaperone activity and its relation to the oligomeric state of the Acr. The manuscript is well written, organized, and presented. Few minor concerns which are mentioned below should be addressed before finalizing the manuscript for indexing.

**Introduction:**

- The last sentence of the first paragraph needs revision. Minor grammatical error is present.

**Results:**

1.  Acr of *Mycobacterium tuberculosis* exists in multiple oligomeric states, as reported earlier, and also mentioned by the authors in the introduction section. As shown in the denaturing gel picture, only prominent SDS resistant dimeric or trimeric forms are visible, other oligomers are present only in minute quantity. Is the dimeric form being the stable and primary oligomeric state of Acr in *M. tb*? The authors should also label gel pictures indicating Molecular weight standards and various oligomers.
2. How the authors calculated the percentage of various oligomers of Acr? Whether they used three gels run with samples purified in different batches or the results given are from a single gel?
3. The sentence starting under the heading “Chaperone activity at 60°C” needs revision. The authors should revise the sentence.

Is the work clearly and accurately presented and does it cite the current literature?

Yes

If applicable, is the statistical analysis and its interpretation appropriate?

Not applicable

Are all the source data underlying the results available to ensure full reproducibility?

Yes

Is the study design appropriate and is the work technically sound?

Yes

Are the conclusions drawn adequately supported by the results?

Yes

Are sufficient details of methods and analysis provided to allow replication by others?

Yes

Reviewer Expertise:

NA

I confirm that I have read this submission and believe that I have an appropriate level of expertise to confirm that it is of an acceptable scientific standard.

# Response

**Published:** 2020-07-02

**Authors:** Gautam Krishnan

**Affiliations:** Birla Institute of Technology & Science, India

The manuscript titled “Prediction of recombinant Mycobacterium tuberculosis α-crystallin oligomer chaperone activity using polynomial graphs” by Gautam Krishnan and Utpal Roy demonstrated the relationship between various higher-order oligomers of Acr and its relation to the prevention of aggregation of insulin B chain as a function of its chaperone activity, predicted using a mathematical polynomial graph. Earlier reports showed that Acr exists in multiple higher-order oligomers, which is related to their chaperone functions. Acr is a vital chaperone protein expressed majorly in the stationary phase as well as in hypoxic conditions. It is required for the survival of M. tb in macrophages; thus, it is a critical virulence effector. The authors utilise the previously known pieces of information and use of polynomial graphs in the prediction of Acr oligomers and its relation to its chaperone activity. They devised a novel way to predict Acr chaperone activity and its relation to the oligomeric state of the Acr. The manuscript is well written, organized, and presented. Few minor concerns which are mentioned below should be addressed before finalizing the manuscript for indexing.

Responses from the authors

Introduction:

The last sentence of the first paragraph needs revision. Minor grammatical error is present.

Acr is active as an oligomer and though its mechanism of action is known to be in the form of a trimer of trimers (Chang et al., 1996) or in the form of a dodecamer (Kennaway et al., 2005; Panda et al., 2017), yet it is difficult to study the mechanism by which it inhibits the aggregation of substrates, either thermally or by dithiothreitol (DTT) induced with insulin.

**Author’s response**: Suggested change►Acr is active in oligomeric state, and though it is known to act as  a  trimer of trimers (Chang et al., 1996) or a dodecamer (Kennaway et al., 2005; Panda et al., 2017), yet it is difficult to study the mechanism by which it inhibits the thermal aggregation or dithiothreitol (DTT)-induced aggregation of insulin.

Results:

 Acr of *Mycobacterium tuberculosis* exists in multiple oligomeric states, as reported earlier, and also mentioned by the authors in the introduction section. As shown in the denaturing gel picture, only prominent SDS resistant dimeric or trimeric forms are visible, other oligomers are present only in minute quantity. Is the dimeric form being the stable and primary oligomeric state of Acr in M. tb? The authors should also label gel pictures indicating Molecular weight standards and various oligomers.

**Author’s response**: The dimer and trimer forms are of the Bovine serine albumin (BSA)standard in Lane 1 and 9 (Figure 1a). These are of the standard. The gel image has been relabelled. The predominant form of *M.tb Acr *is nonamer to 12-mers which could be an aggregation of trimers or dimers. Chang et al, 1996 showed that the repeating unit is a trimer and a trimer of trimers forms a nonamer. We need to do more experimentation to verify the same.

The re-labelling of the images has been done as suggested by the Honorable reviewer. As can be seen in Figure lanes 3,7 and 8 there is a proportion of 9 to 12 mers in the non-gel filtered samples as compared to higher proportion of 12 mers in lanes 4,5 and 6.

How the authors calculated the percentage of various oligomers of Acr? Whether they used three gels run with samples purified in different batches or the results given are from a single gel?

**Author’s Response**:The percentage of various oligomers was calculated using software called Image J. This was accomplished by measuring the intensity of each band calculating the total intensity and dividing the intensity of each band by the total intensity to obtain the proportion. The molecular weight of oligomers was obtained by comparing with the standard Bovine serum albumin standards. The results are from a single gel. All batches were analyzed similarly and showed a range of oligomers concentrations which could explain the relation between higher oligomer ratio and activity.

The sentence starting under the heading “Chaperone activity at 60°C” needs revision. The authors should revise the sentence.

**Author’s Response**:According to the suggestion heading has been changed to ‘Chaperone activity of Recombinant Acr at higher temperature’

# Peer review: Reviewer response for version 1

**DOI:** 10.5256/f1000research.17837.r40682

**Published:** 2019-01-07

**Authors:** Anjan Purkayastha

**Affiliations:** 1 Leidos Biomedical Research, Reston, VA, USA

The Acr protein is a chaperone that prevents misfolding of proteins in latent *M. tuberculosis*.  Characterizing the precise molecular mechanism of action of Acr, however, remains a challenge. The authors, Krishan and Roy, have used a novel means of estimating the degree of oligomerization using polynomial graphs. The authors make two key observations: the coverage of the insulin B molecules is influenced by (a)the degree of Acr oligomerization and (b)the ratio of oligomers. 

 This work lays the groundwork for further experiments in understanding the chaperoning activity of Acr at a molecular level.

Is the work clearly and accurately presented and does it cite the current literature?

Yes

If applicable, is the statistical analysis and its interpretation appropriate?

Yes

Are all the source data underlying the results available to ensure full reproducibility?

Yes

Is the study design appropriate and is the work technically sound?

Yes

Are the conclusions drawn adequately supported by the results?

Yes

Are sufficient details of methods and analysis provided to allow replication by others?

Yes

Reviewer Expertise:

Infectious disease, bioinformatics

I confirm that I have read this submission and believe that I have an appropriate level of expertise to confirm that it is of an acceptable scientific standard.
